# Role of macrophage-mediated Toll-like receptor 4–interleukin-1R signaling in ectopic tongue pain associated with tooth pulp inflammation

**DOI:** 10.1186/s12974-020-01995-y

**Published:** 2020-10-21

**Authors:** Kohei Kanno, Kohei Shimizu, Masamichi Shinoda, Makoto Hayashi, Osamu Takeichi, Koichi Iwata

**Affiliations:** 1 Department of Endodontics, Nihon University School of Dentistry, 1-8-13 Kandasurugadai, Chiyoda-ku, Tokyo, 101-8310 Japan; 2 Division of Advanced Dental Treatment, Dental Research Center, Nihon University School of Dentistry, Tokyo, Japan; 3 Department of Physiology, Nihon University School of Dentistry, Tokyo, Japan; 4 Division of Functional Morphology, Dental Research Center, Nihon University School of Dentistry, Tokyo, Japan

**Keywords:** Intercellular signaling, Ectopic tongue pain, Toll-like receptors, Interleukin-1β, Transient receptor potential vanilloid 1, Cytokine receptors, Chemokine, Hyperalgesia, Trigeminal ganglion

## Abstract

**Background:**

The existence of referred pain and ectopic paresthesia caused by tooth pulp inflammation may make definitive diagnosis difficult and cause misdiagnosis or mistreatment; thus, elucidation of that molecular mechanism is urgent. In the present study, we investigated the mechanisms underlying ectopic pain, especially tongue hyperalgesia, after tooth pulp inflammation.

**Methods:**

A rat model with mandibular first molar tooth pulp exposure was employed. Tooth pulp exposure-induced heat and mechanical-evoked tongue hypersensitivity was measured, and immunohistochemical staining for Iba1, a marker of active macrophages, IL-1β, IL-1 type I receptor (IL-1RΙ), and toll-like receptor 4 in the trigeminal ganglion was performed. In addition, we investigated the effects of injections of liposomal clodronate Clophosome-A (LCCA), a selective macrophage depletion agent, lipopolysaccharide from *Rhodobacter sphaeroides* (LPS-RS, a toll-like receptor 4 antagonist), IL-1β, or heat shock protein 70 (Hsp70, a selective agonist of toll-like receptor 4), to examine changes in tongue hypersensitivity and in the regulation of IL-1RΙ, toll-like receptor 4, and transient receptor potential vanilloid 1 (TRPV1) biosynthesis.

**Results:**

At day 1 after tooth pulp exposure, obvious tooth pulp inflammation was observed. Tooth pulp exposure-induced heat and mechanical tongue hypersensitivity was observed from days 1 to 3 after tooth pulp exposure. The production of IL-1β in activated macrophages and toll-like receptor 4 and IL-1RΙ expression were significantly increased in trigeminal ganglion neurons innervating the tongue following tooth pulp exposure. Intra-trigeminal ganglion injection of LCCA significantly suppressed tongue hypersensitivity; however, toll-like receptor 4 and IL-1RΙ expression in trigeminal ganglion neurons innervating the tongue was not significantly altered. Intra-trigeminal ganglion injection of LPS-RS significantly suppressed tongue hypersensitivity and reduced IL-1RΙ expression in the trigeminal ganglion neurons innervating the tongue following tooth pulp exposure. Intra-trigeminal ganglion injection of recombinant Hsp70 significantly promoted tongue hypersensitivity and increased IL-1RI expression in trigeminal ganglion neurons innervating the tongue in naive rats. Furthermore, intra-trigeminal ganglion injection of recombinant IL-1β led to tongue hypersensitivity and enhanced TRPV1 expression in trigeminal ganglion neurons innervating the tongue in naive rats.

**Conclusions:**

The present findings suggest that the neuron-macrophage interaction mediated by toll-like receptor 4 and IL-1RI activation in trigeminal ganglion neurons affects the pathogenesis of abnormal tongue pain following tooth pulp inflammation via IL-1RI and TRPV1 signaling in the trigeminal ganglion. Further research may contribute to the establishment of new therapeutic and diagnostic methods.

## Background

It is well known that orofacial paresthesia or ectopic referred pain sometimes occurs as secondary hyperalgesia associated with tooth pulp inflammation [1–4]. Persistent orofacial pain following trigeminal nerve injury or orofacial inflammation causes a variety of motor and sensory disorders in the orofacial area, including masticatory deficits and/or dysphagia [5]. Recent pain studies indicated that one of the factors that can induce such symptoms is long-term sensitization of peripheral nerves and enhancement of nerve excitability, resulting in hypersensitivity of the peripheral and central nervous system [6–9]. However, the detailed mechanism of how the peripheral nervous system is associated with the development of orofacial-referred pain following tooth pulp inflammation remains unclear. The existence of referred pain and ectopic paresthesia caused by tooth pulp inflammation may make definitive diagnosis difficult, leading to misdiagnosis or mistreatment. Therefore, elucidation of this molecular mechanism is urgent.

In the trigeminal ganglion, not only are the cell bodies of primary neurons present, but also satellite glial cells, macrophages and lymphocytes. Previous studies and our reports indicated that these non-neuronal cells are involved in increased excitability of primary sensory neurons through various signal transductions [10–12]. Therefore, in the present study, we focused on macrophages, one of the non-neuronal cells found in the trigeminal ganglion. Macrophages are a major source of pro-inflammatory cytokines, including interleukin-1β (IL-1β) [10, 13–15], a prototypic pro-inflammatory cytokine that induces cellular signal transduction via the IL-1 type I receptor (IL-1RΙ) and regulates the sensation of pain and inflammatory condition [16, 17]. Following peripheral tissue inflammation, the accumulated macrophages release IL-1β in the dorsal root ganglion, leading to secondary hyperalgesia [4]. However, despite evidence suggesting the involvement of microglia-neuron interaction in this process via pro-inflammatory cytokines [10–12], the detailed mechanism underlying the observed effects in the trigeminal ganglion mediated by the accumulated macrophages associated with tooth pulp inflammation is not fully understood.

Toll-like receptors mediate signaling in response to various pathogen-associated molecular patterns [18, 19]. Following tissue injury, inflammation, or cellular stress, toll-like receptors recognize danger- and pathogen-associated molecular patterns that act as endogenous ligands [19]. Toll-like receptors expressed in primary sensory neurons detect danger- and pathogen-associated molecular patterns released following tissue damage or cellular stress and assist in modulating neuronal excitation [20–22]. Peripheral orofacial inflammation leads to enhanced toll-like receptor 4 expression in the trigeminal ganglion, in turn inducing ectopic orofacial pain [21–23]. It is well known that heat shock protein 70 (Hsp70) is one of the major ligands for toll-like receptor 4. Hsp70 has also been reported to be expressed in the pulp due to tooth trauma and pulpitis [22, 24]. In previous studies, we reported that Hsp70s expression is increased in the inflamed pulp tissue following pulpitis and that Hsp70s are axonally transported to trigeminal ganglion cell bodies innervating the inflamed pulp. Transported Hsp70s are released extracellularly from trigeminal ganglion cell bodies and bind to toll-like receptor 4 expressed on trigeminal ganglion cell bodies that are innervating the tongue via paracrine signaling, inducing tongue hyperalgesia [21]. Other studies indicated that toll-like receptor 4-signaling upregulates IL-1β and IL-1RI expression and promotes alveolar macrophage pyroptosis and lung inflammation through an autocrine mechanism [25]. Additionally, peripheral inflammation or nerve injury leads to enhanced IL-1RΙ expression in trigeminal ganglion, which has a pivotal role in facilitating trigeminal ganglion neuronal excitability, and thereby contributes to orofacial hyperalgesia development [16, 26, 27]. Based on this evidence, we hypothesized that toll-like receptor 4 and IL-1RΙ expression is increased in the trigeminal ganglion following tooth pulp inflammation and involved in the enhancement of neuronal excitability in trigeminal ganglion.

Transient receptor potential vanilloid 1 (TRPV1), which is activated by noxious heat (> 43 °C), low extracellular pH (< 6.5), and some irritants, is also considered to contribute to mechanical nociception [28]. TRPV1 expression in sensory neurons increases following the upregulation of IL-1β production by activated satellite glial cells [29]. Hence, it is likely that the expression of danger-associated molecular patterns induced by the inflammation of the orofacial organ, such as tooth pulp inflammation induced by mandibular first molar tooth pulp exposure, also induces TRPV1 and IL-1RΙ expression via toll-like receptor 4 signaling in trigeminal ganglion neurons.

Accordingly, we hypothesized that tooth pulp inflammation induces tongue referred pain as a symptom of orofacial ectopic pain. We further hypothesized that cytokines produced by macrophages are involved in this process and that the macrophage-neuron interaction contributes to the tongue hyperalgesia following tooth pulp exposure. To examine this hypothesis, we investigated tooth pulp exposure-induced heat and mechanical-evoked nocifensive reflex of the tongue in a rat model of the mandibular first molar tooth pulp exposure and then performed immunohistochemical staining for Iba1, a marker of active macrophages, IL-1β, IL-1RΙ, and toll-like receptor 4 in the trigeminal ganglion. In addition, we investigated the effects of injections of liposomal clodronate Clophosome-A (LCCA), a selective macrophage depletion agent, lipopolysaccharide from *Rhodobacter sphaeroides* (LPS-RS, a toll-like receptor 4 antagonist), IL-1β, or heat shock protein 70 (Hsp70, a selective agonist of toll-like receptor 4) to examine the change in tongue hypersensitivity and in the regulation of IL-1RΙ, toll-like receptor 4, and TRPV1 biosynthesis.

## Methods

### Animals

**Fig. 1:**
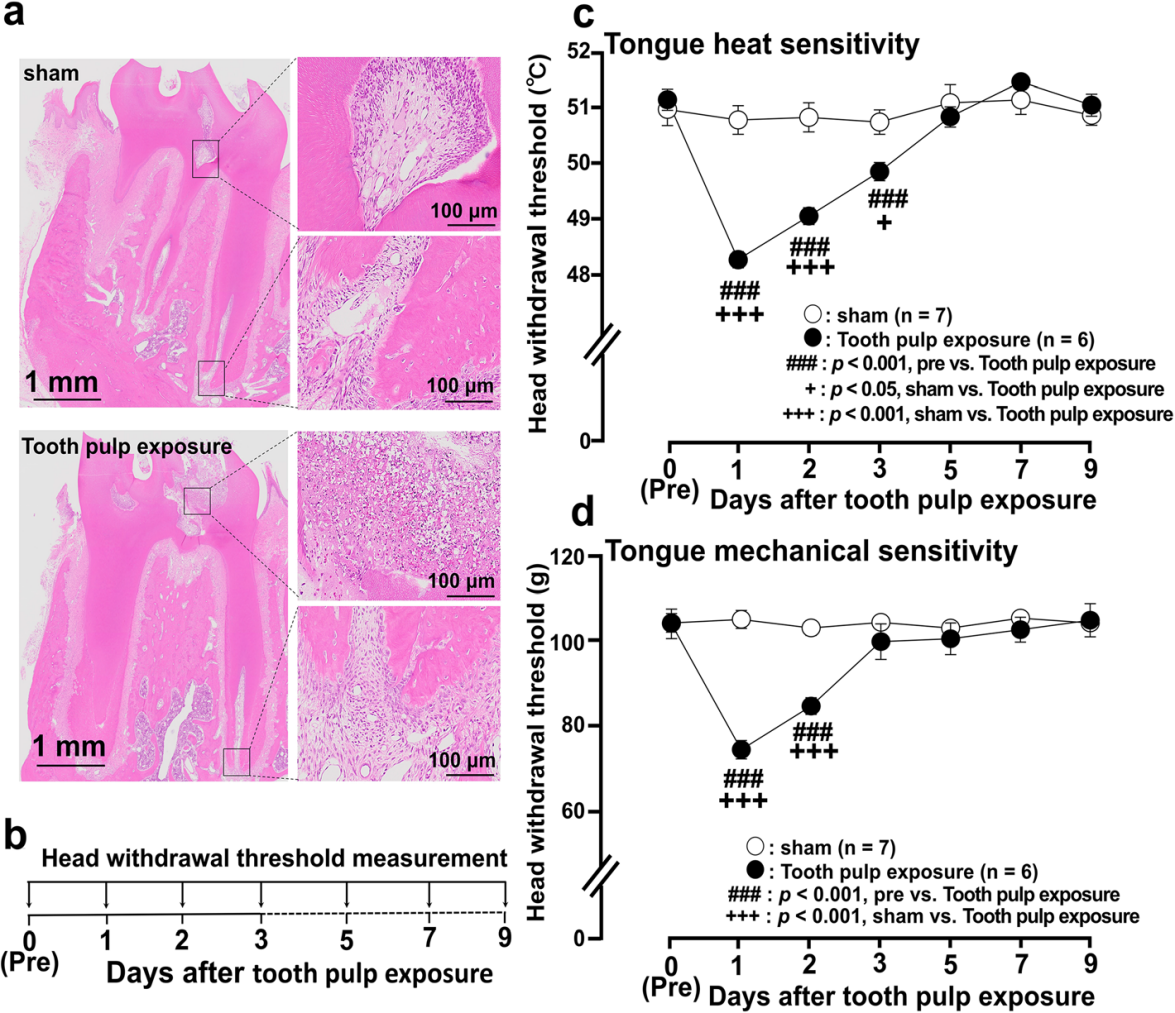
Photomicrographs of tooth pulp inflammation and nocifensive reflex to mechanical or heat stimulation of the tongue following tooth pulp exposure. **a** Photomicrographs of histological sections of the mandibular first molar 1 day after tooth pulp exposure. **b** Time course of behavioral tests. **c**, **d** Head withdrawal threshold to heat (**c**) or mechanical (**d**) stimulation of the ipsilateral tongue following tooth pulp exposure. Group comparisons were performed using two-way repeated-measures ANOVA followed by *Tukey*–*Kramer* post hoc tests. Data are expressed as the mean ± SEM

**Fig. 2:**
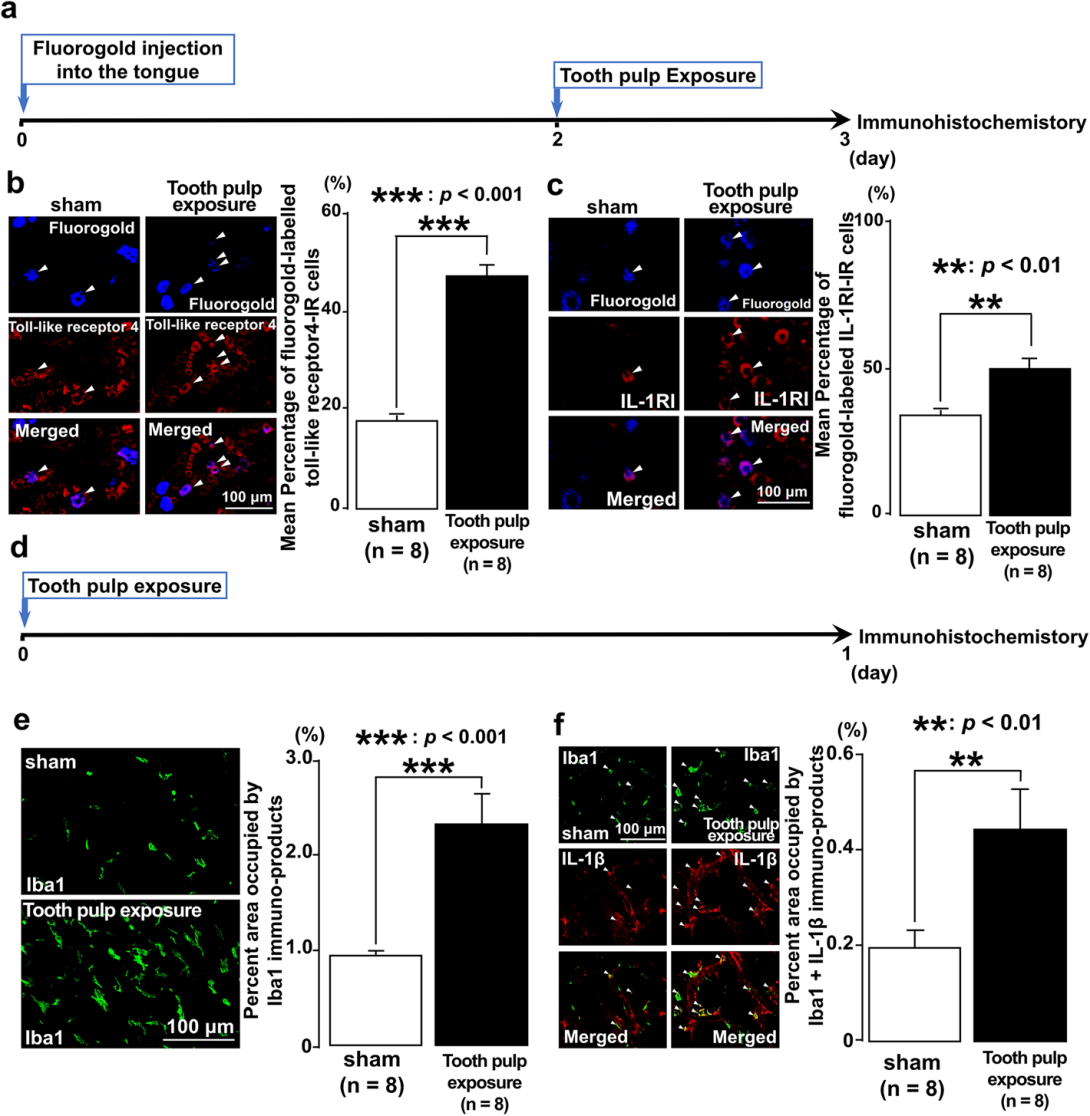
Toll-like receptor 4, IL-1RI, Iba1, and IL-1β expression in the trigeminal ganglion following tooth pulp exposure. **a** Time course of the experimental procedure. **b** Fluorogold-labeling of toll-like receptor 4-IR cells in the trigeminal ganglion following tooth pulp exposure. The arrowheads indicate fluorogold-labeled toll-like receptor 4-IR cells. Scale bar, 100 μm. The number of each IR cell type in the trigeminal ganglion of the third branch region was calculated by using the following formula: fluorogold-labeled toll-like receptor 4-IR cells/fluorogold-labeled cells × 100%. **c** Fluorogold-labeled IL-1RI-IR cells in the trigeminal ganglion following tooth pulp exposure. The arrowheads indicate fluorogold-labeled IL-1RI-IR cells. Scale bar, 100 μm. The number of each IR cell type in the trigeminal ganglion of the third branch region was calculated by using the following formula: fluorogold-labeled IL-1RI-IR cells/fluorogold-labeled cells × 100%. **d** Time course of the experimental procedure. **e** Iba1-IR cells in the trigeminal ganglion following tooth pulp exposure. Scale bar, 100 μm. The percentage of the area occupied by immuno-products was calculated. **f** Iba1-IR cells expressing IL-1β in the trigeminal ganglion following tooth pulp exposure. The arrowheads indicate Iba1-IR cells expressing IL-1β. Scale bar, 100 μm. The number of each IR cell type in the trigeminal ganglion of the third branch region was calculated by using the following formula: immuno-products of Iba1 + IL-1β/Iba1 × 100%. The Student’s *t* test was employed for the comparison of the percentages of positive cells between groups. Data are expressed as the mean ± SEM

**Fig. 3:**
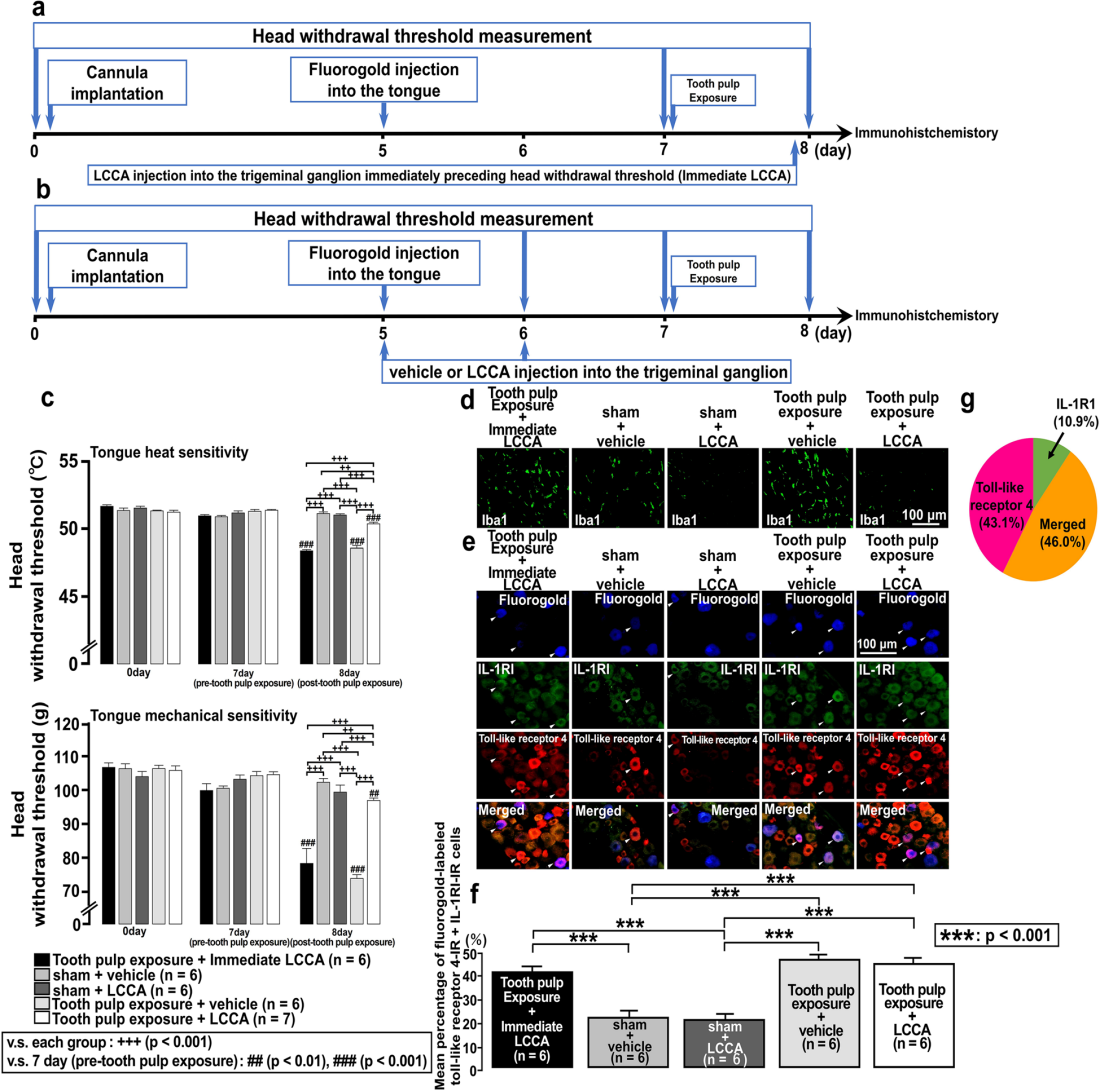
Effect of liposomal clodronate Clophosome-A injection into the trigeminal ganglion in rats with tooth pulp inflammation. **a** Time course of the experimental procedure for the group administered liposomal clodronate Clophosome-A (LCCA) into the trigeminal ganglion immediately preceding head withdrawal threshold measurement in rats with tooth pulp exposure (Tooth pulp exposure + Immediate LCCA). **b** Time course of the experimental procedure for the groups administered vehicle or LCCA into the trigeminal ganglion of rats with sham or tooth pulp exposure procedures. **c** Head withdrawal threshold to heat or mechanical stimulation of the ipsilateral tongue following vehicle or LCCA injection into the trigeminal ganglion of rats with sham or tooth pulp exposure procedures. The two-way repeated-measures ANOVA followed by Tukey–Kramer post hoc tests were used to compare head withdrawal thresholds between groups. **d** Photographs of the change in Iba1 expression in the trigeminal ganglion following administration of vehicle or LCCA into the trigeminal ganglion of rats with sham or tooth pulp exposure procedures. Scale bar, 100 μm. **e** Fluorogold-labeled toll-like receptor 4-IR and IL-1RI-IR cells in the trigeminal ganglion following administration of vehicle or LCCA into the trigeminal ganglion of rats with sham or tooth pulp exposure procedure. The arrowheads indicate fluorogold-labeled toll-like receptor 4-IR and IL-1RI-IR cells. Scale bar, 100 μm. **f** The number of each IR cell type in the trigeminal ganglion of the third branch region was calculated by using the following formula: fluorogold-labeled toll-like receptor 4-IR and IL-1RI-IR cells/fluorogold-labeled cells × 100%. The one-way repeated-measures ANOVA followed by Tukey’s post hoc test was employed for the comparison of the percentages of positive cells between groups. Data are expressed as the mean ± SEM. **g** The pie graph for the percentages of toll-like receptor 4-IR, IL-1RI-IR, and merged cells in the trigeminal ganglion

**Fig. 4:**
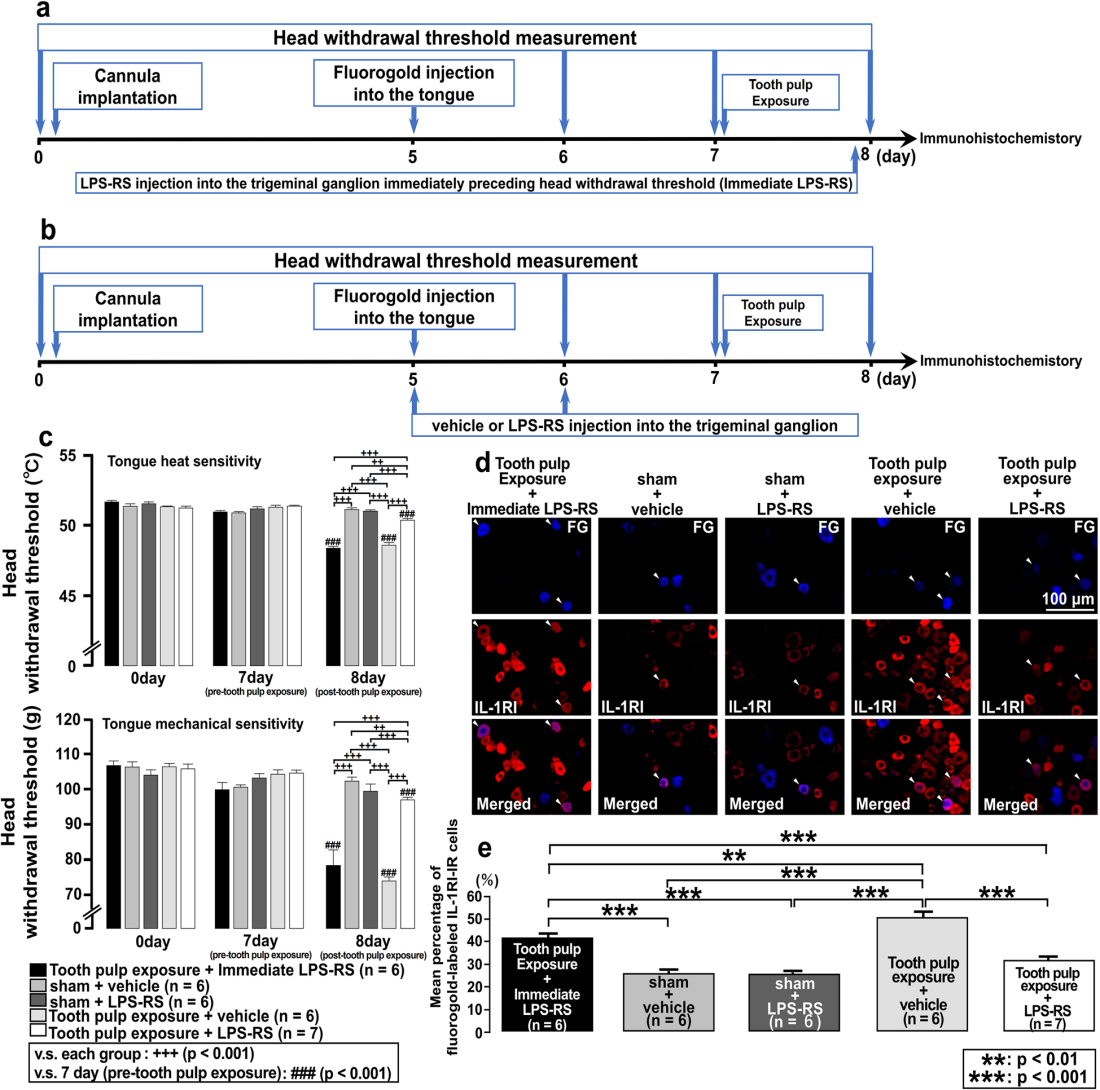
Effect of LPS-RS injection into the trigeminal ganglion in rats with tooth pulp inflammation. **a** Time course of the experimental procedure for the group administered LPS-RS into the trigeminal ganglion immediately preceding head withdrawal threshold measurement in rats with tooth pulp exposure (Tooth pulp exposure + Immediate LPS-RS). **b** Time course of the experimental procedure for the groups administered vehicle or LPS-RS into the trigeminal ganglion of rats with sham or tooth pulp exposure procedures. **c** The head withdrawal threshold to heat or mechanical stimulation of the ipsilateral tongue following administration of vehicle or LPS-RS into the trigeminal ganglion of rats with sham or tooth pulp exposure procedures. The two-way repeated-measures ANOVA followed by Tukey–Kramer post hoc tests were used to compare head withdrawal thresholds between groups. **d** Fluorogold-labeled IL-1RI-IR cells in the trigeminal ganglion following administration of vehicle or LPS-RS into the trigeminal ganglion of rats with sham or tooth pulp exposure procedures. The arrowheads indicate fluorogold-labeled IL-1RI-IR cells. Scale bar, 100 μm. **e** The number of each IR cell type in the trigeminal ganglion of the third branch region was calculated by using the following formula: fluorogold-labeled IL-1RI-IR cells/fluorogold-labeled cells × 100%. The one-way repeated-measures ANOVA followed by Tukey–Kramer post hoc test was employed for the comparison of the percentages of positive cells between groups. Data are expressed as the mean ± SEM

**Fig. 5:**
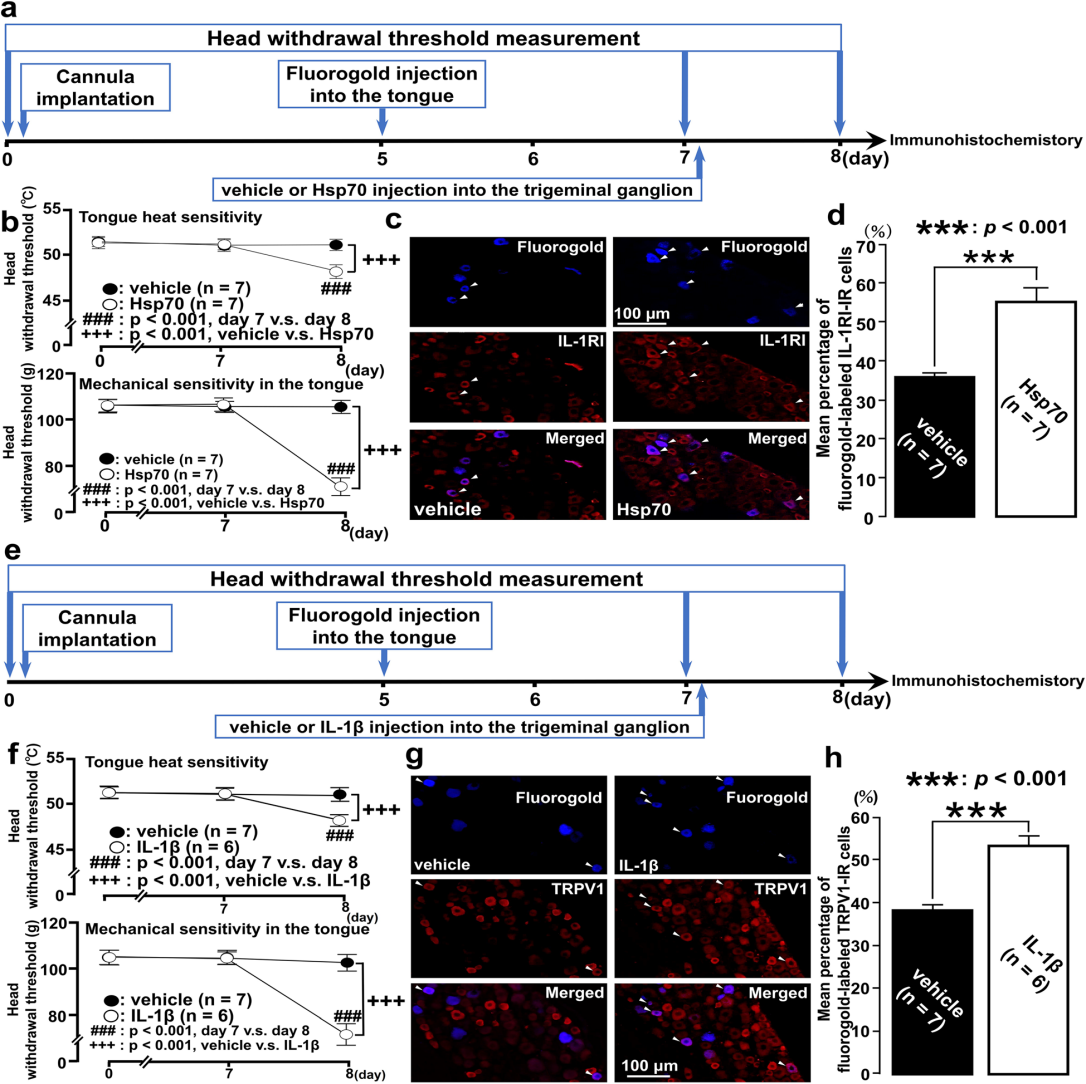
Effects of Hsp70 or IL-1β injection in the trigeminal ganglion. **a** Time course of the experimental procedure for the groups administered vehicle or Hsp70 into the trigeminal ganglion. **b** Head withdrawal threshold to heat or mechanical stimulation of the ipsilateral tongue following administration of Hsp70 into the trigeminal ganglion. Two-way repeated-measures ANOVA followed by Tukey’s post hoc tests were used to compare head withdrawal thresholds between groups. **c** Fluorogold-labeled IL-1RI-IR cells following Hsp70 injection into the trigeminal ganglion. The arrowheads indicate fluorogold-labeled IL-1RI-IR cells. Scale bar, 100 μm. **d** The number of each IR cell type in the trigeminal ganglion of the third branch region was calculated using the following formula: fluorogold-labeled IL-1RI-IR cells/fluorogold-labeled cells × 100%. The Student’s *t* test was employed for the comparison of percentages of positive cell between groups. **e** Time course of the experimental procedure for the groups administered vehicle or Hsp70 into the trigeminal ganglion. **f** Head withdrawal threshold to heat or mechanical stimulation of the ipsilateral tongue following IL-1β injection into the trigeminal ganglion. Two-way repeated-measures ANOVA followed by Tukey–Kramer post hoc tests were used to compare head withdrawal thresholds between groups. **g**, **h** Fluorogold-labeled TRPV1-IR cells following IL-1β injection into the trigeminal ganglion. The arrowheads indicate fluorogold-labeled TRPV1-IR cells. Scale bar, 100 μm. The number of each IR cell type in the trigeminal ganglion of the third branch region was calculated by using the following formula: fluorogold-labeled TRPV1-IR cells/fluorogold-labeled cells × 100%. The Student’s *t* test was employed for the comparison of percentages of positive cells between groups. Data are expressed as the mean ± SEM

We utilized male Sprague-Dawley rats (*n* = 167, Japan SLC, Shizuoka, Japan) weighing 250–350 g. Rats were housed at a stable temperature (23 °C) with a 12 h light to dark cycle (7:00–19:00:19:00–7:00). Animals were housed individually in transparent polycarbonate cages (length, 48 cm; width, 26.5 cm; height, 21 cm) with paper shavings as bedding and were allowed ad libitum access to food and water. The study was approved by the Nihon University Animal Experiment Committee (protocol numbers AP17D021, AP19DEN009-1, and AP19DEN009-2). The study was conducted according to the National Institutes of Health guidelines on laboratory animal management and use and based the previous pain study reports [30]. These experiments were minimized the number of rats for the statistics. The study complied with ARRIVE guidelines. The animal groups were as follows: head withdrawal threshold measurement of tongue heat and mechanical sensitivity (Fig. 1, sham: *n* = 7, tooth pulp exposure: *n* = 6); toll-like receptor 4, IL-1RI, Iba1, and IL-1β expression in the trigeminal ganglion following tooth pulp exposure (Fig. 2, sham: *n* = 8, tooth pulp exposure: *n* = 8 each); effect of LCCA injection into the trigeminal ganglion following tooth pulp exposure (Fig. 3, tooth pulp exposure + LCCA immediately preceding head withdrawal threshold measurement: *n* = 6, sham + vehicle: *n* = 6, sham + LCCA: *n* = 6, tooth pulp exposure + vehicle: *n* = 6, tooth pulp exposure + LCCA: *n* = 7); effect of LPS-RS injection into the trigeminal ganglion following tooth pulp exposure (Fig. 4, tooth pulp exposure + LPS-RS immediately preceding head withdrawal threshold measurement: *n* = 6, sham + vehicle: *n* = 6, sham + LPS-RS: *n* = 6, tooth pulp exposure + vehicle: *n* = 6, tooth pulp exposure + LPS-RS: *n* = 7); and effect of Hsp70 or IL-1β injection into the trigeminal ganglion on head withdrawal threshold measurement and IL-1RI-IR or TRPV1-IR cell expression in the trigeminal ganglion, respectively (Fig. 5b–d, vehicle: *n* = 7, Hsp70: *n* = 7, Fig. 5f–h, vehicle: *n* = 7, IL-1β: *n* = 6).

### The procedure of anesthesia

For the head withdrawal threshold measurement of tongue heat and mechanical sensitivity, fluorogold injection into the tongue, and the administration of the drug (LCCA, LPS-RS, Hsp70, and IL-1β) into the trigeminal ganglion, we used light inhalant anesthesia using a mixture of isoflurane (2%, Mylan, Canonsburg, PA) and oxygen. For the tooth pulp exposure of the rat mandibular first molar, cannula implantation for the administration of the drug into the trigeminal ganglion, and perfusion for the immunohistochemistry, we used light inhalant anesthesia using a mixture of isoflurane and oxygen, and then deeply anesthetized with an intraperitoneal injection of butorphanol (2.5 mg/kg, Meiji Seika Pharma, Tokyo, Japan), medetomidine (0.375 mg/kg, Zenoaq, Fukushima, Japan), and midazolam (2.0 mg/kg, Sandoz, Tokyo, Japan) dissolved in saline (three-mixed anesthetic).

### Histochemical analysis of the dental pulp after tooth pulp exposure of the rat mandibular first molar

Rats were anesthetized with light inhalant anesthesia using a mixture of isoflurane and oxygen, and then deeply anesthetized with an intraperitoneal injection of three-mixed anesthetic. The procedure of mandibular first molar exposure was performed as referenced in a previous report [22, 31]. After anesthesia, the rats were then placed on a warm mat (37 °C) in the supine position to perform the surgical procedure. The rat’s mouth was gently opened with metal tweezers and the left maxillary first molar was drilled open using a low-speed dental drill with a round tungsten carbide bur under water cooling. The whole enamel and dentin on occlusal surfaces were grinded off to induce a type III injury and subsequent irreversible pulpitis with exposure of the pulp to the oral environment [31, 32]. The exposed pulp was kept open and rats were conveyed back to their cage. Sham animals were subjected to the same process of anesthesia but without any other intervention. One day after tooth pulp exposure, the rats (*n* = 3) were anesthetized with light inhalant anesthesia using a mixture of isoflurane and oxygen, and then deeply anesthetized with an intraperitoneal injection of three-mixed anesthetic and transcardially perfused as previously described [21, 33, 34]. The mandibular jaw was removed and decalcified with EDTA for 7 days, 3 μm sections were made from the extracted tooth samples of the tooth pulp exposure and sham, and the sections were stained with hematoxylin and eosin to examine if tooth pulp inflammation has developed.

### Head withdrawal threshold measurements

Head withdrawal threshold was measured under light anesthesia with 2% isoflurane and oxygen for mechanical and heat stimulation of the tongue ipsilateral to tooth pulp exposure (3 mm behind the tip of the tongue). Head withdrawal threshold measurements were performed on days 1, 2, 3, 5, 7, and 9 after tooth pulp exposure as previously described [13]. Briefly, the lower jaw was gently pulled with plastic strings to hold the mouth open and then heat stimulation (35–60 °C; 1 °C/s; cutoff, 60 °C) or mechanical stimulation (0–130 g; 10 g/s; cutoff, 130 g) was applied to the lateral tongue edge. Flat-tip forceps (4 mm^2^; Panlab S.L., Barcelona, Spain) or a contact heat probe (9 mm^2^; Intercross, Tokyo, Japan) was used for mechanical or heat stimulation, respectively. Heat or mechanical stimulation was applied three times at 5-min intervals, and the mean value of the Head withdrawal thresholds was calculated. Changes in mean head withdrawal threshold values over time were measured in tooth pulp exposure or sham rats. Baseline of head withdrawal threshold levels was measured before sham or tooth pulp exposure procedure [21].

### Immunohistochemistry

Trigeminal ganglion neurons innervating the tongue were visualized using fluorogold (a retrograde tracer, Fluorochrome, Denver, CO) which was injected into the tongue. In rats anesthetized with 2% isoflurane, 10.0 μL of 4% fluorogold dissolved in saline was injected into the tongue ipsilateral to tooth pulp exposure. Tooth pulp exposure was performed under anesthesia mentioned above on the second day after fluorogold injection. On day 1 after tooth pulp exposure, and after head withdrawal threshold measurement was completed, the rats were lightly anesthetized using a mixture of 2% isoflurane and oxygen, deeply anesthetized with the three-mixed anesthetic, and transcardially perfused as previously described [33]. Trigeminal ganglion sections were prepared (15-μm-thick), mounted on microscope slides (Matsunami, Tokyo, Japan), and blocked to avoid non-specific binding of antibodies. Sections were then incubated in a solution of rabbit anti-toll-like receptor 4 (1:1000, ab13556; Abcam, Cambridge, UK), rabbit anti-IL1RI (1:500, SC-689; Santa Cruz Biotechnology, Dallas, TX), mouse anti-IL-1β (1:800, ab8320; Abcam), rabbit anti-Iba1 (1:2000, 019-19741; Wako, Shiga, Japan), mouse anti-IL1RI (1:500, ab40101; Abcam), or guinea-pig anti-TRPV1 (1:500, AB10295; Abcam) polyclonal antibody. The sections were then incubated in the secondary antibody solution of Alexa Fluor 568-conjugated goat anti-rabbit IgG, Alexa Fluor 568-conjugated goat anti-mouse IgG, Alexa Fluor 488-conjugated goat anti-mouse IgG, and/or Alexa Fluor 568-conjugated goat anti-guinea pig IgG (all 1:200 in 0.01 M phosphate-buffered saline; Thermo Fisher Scientific, Waltham, MA). Following the application of mounting medium, trigeminal ganglion sections were cover-slipped, and the immunoreactive (IR) cells were observed by fluorescence microscopy using a BZ-9000 system (Keyence, Osaka, Japan). Double- or triple-labeled cells (fluorogold and toll-like receptor 4; fluorogold and IL-1RI; Iba1 and IL-1β; fluorogold, toll-like receptor 4, and IL-1RI; fluorogold and TRPV1) were recognized as those exhibiting co-expression of respective markers. Next, we precisely counted the number of cells and performed analysis using a computer-assisted imaging analysis system (BZ-X analyzer, Keyence). Cells displaying the signal over twice the saturation level compared to the mean background signal were identified as positive. Fluorogold-labeled IR cells in the third branch region of the trigeminal ganglion were counted. The equations used to calculate and analyze IR cells in the trigeminal ganglion of the third branch region are given in the legends to Figs. 2, 3, 4, 5.

### Drug injection into the trigeminal ganglion

Rats were lightly anesthetized using a mixture of 2% isoflurane and oxygen, and then deeply anesthetized with the three-mixed anesthetic. A midline skin incision (2-cm-long) was made from head to neck and a small hole (1 mm in diameter) was made in the skull above the trigeminal ganglion. A guide cannula was inserted into the brain through the hole to reach the trigeminal ganglion; the cannula tip was placed 9 mm below the skull surface. Next, as previously reported, the cannulation procedure was performed on the skull surface [34]. Phosphate-buffered saline (1 μL/day), LPS-RS (0.1 mM, 1 μL/day; InvivoGen, San Diego, CA), LCCA (7 mg/mL, 1 μL/day; F70101CA, FormuMax Scientific, Sunnyvale, CA), or plain control liposomes for LCCA (Cont-LCCA; 20 mM, 1 μL/day; F70101-A, FormuMax Scientific) was injected into the trigeminal ganglion once daily for two successive days. The dosages of LPS-RS and LCCA were based on previous reports [21, 33]. Then, we measured head withdrawal threshold and performed immunohistochemistry to investigate each drug’s effect on tongue hypersensitivity following tooth pulp exposure.

### Injection of Hsp70 or IL-1β into the trigeminal ganglion

Rats were lightly anesthetized using a mixture of 2% isoflurane and oxygen. Recombinant human Hsp70/HSPA1A (1 mL, 1 mg/mL, AP-100-100, R&D Systems, Minneapolis, MN) or recombinant mouse IL-1β protein (1 mL, 1 mg/mL, AB9723, Abcam) was injected into the left trigeminal ganglion using a guide cannula on day 2 following fluorogold injection. On day 1 after injection, head withdrawal threshold to heat and mechanical stimulation of the tongue was measured, then rats were transcardially perfused as previously described [21, 33, 34]. Then, we prepared trigeminal ganglion sections, mounted them on microscopic slides, and incubated them with rabbit anti-IL1RI or guinea pig anti-TRPV1 polyclonal antibody solutions. Saline was also injected as vehicle control.

### Statistical analysis

Data are presented as the mean ± standard error of the mean (SEM). Head withdrawal thresholds between the groups were compared using two-way repeated-measures analysis of variance (ANOVA) followed by Tukey’s or Tukey–Kramer post hoc tests. The percentages of positive cells between the groups were compared using the Student’s *t* test or the one-way ANOVA followed by Tukey or Tukey–Kramer post hoc tests. All statistical analyses were conducted with a significance level of *α* = 0.05 (*p* < 0.05).

## Results

### Tooth pulp inflammation and nocifensive reflexes against heat and mechanical stimulation of the tongue

Previous studies indicated that tongue hyperalgesia is induced from day 1 following tooth pulp exposure or tooth pulp inflammation [21, 22]. Therefore, to examine if tooth pulp inflammation had developed under the present experimental condition, hematoxylin and eosin staining was performed on the extracted teeth following tooth pulp exposure or sham procedure first. Numerous inflammatory cells were observed in the coronal, but not in the apical pulp in rats on day 1 after tooth pulp exposure (Fig. 1a). Next, to observe the changes in nocifensive reflexes against heat or mechanical stimulation of the tongue following tooth pulp exposure, a time course analysis of head withdrawal threshold measurements was performed. The tooth pulp exposure to heat or mechanical stimulation of the tongue ipsilateral to the tooth pulp exposure was significantly reduced after tooth pulp exposure compared to that in pre- tooth pulp exposure (day 0) on day 1 (heat: *p* = 0.001, mechanical: *p* = 0.001), day 2 (heat: *p* = 0.001, mechanical: *p* = 0.001), and day 3 (heat: *p* = 0.001) (Fig. 1b–d). Head withdrawal threshold was also significantly reduced after tooth pulp exposure compared to that in sham-treated rats on day 1 (heat: *p* = 0.001, mechanical: *p* = 0.001), day 2 (heat: *p* = 0.001, mechanical: *p* = 0.001), and day 3 (heat: *p* = 0.039). No changes in head withdrawal threshold were observed in sham rats (Fig. 1c, d). These results indicate that tooth pulp inflammation and tongue hyperalgesia are developed following tooth pulp exposure under the present experimental condition.

### Expression of toll-like receptor 4, IL-1RI, Iba1, and IL-1β in trigeminal ganglion after tooth pulp exposure

Previous study suggested that the expression of toll-like receptor 4 and IL-1β are increased on day 1 following tooth pulp exposure [22]. On the other hand, macrophage accumulations are caused in the trigeminal ganglion followed orofacial inflammation, and the accumulated macrophages secretes neuromodulators to drive ectopic and chronic pain by inducing neuronal and synaptic plasticity [8–10, 12]. To examine the changes in expression of toll-like receptor 4, IL-1RI, Iba1, and IL-1β in trigeminal ganglion after tooth pulp exposure, immunohistochemistry was performed following operations of sham or tooth pulp exposure. We observed an increased number of fluorogold-labeled toll-like receptor 4-IR and fluorogold-labeled IL-1RI-IR cells in trigeminal ganglion on day 1 after procedure of tooth pulp exposure. Furthermore, the average percentages of fluorogold labeled toll-like receptor 4-IR and IL-1RI-IR cells were significantly greater in rats with tooth pulp exposure (toll-like receptor 4-IR: *p* = 0.001, IL-1RI-IR: *p* = 0.004) than those in sham-rats (Fig. 2a–c). More Iba1-IR and Iba1-IL-1β-IR cells were also observed in tooth pulp exposure-rats (Fig. 2d–f). In addition, the areas occupied by Iba1 and Iba1-IL-1β immuno-products were significantly larger in rats with tooth pulp exposure (Iba1: *p* = 0.001, Iba1-IL-1β: *p* = 0.005) than those in sham-rats (Fig. 2d–f). These findings indicate that tooth pulp exposure induces increment of the expression of toll-like receptor 4 and IL-1RI in trigeminal ganglion and also suggested that tooth pulp exposure leads to the macrophage accumulations in the trigeminal ganglion and overexpression of IL-1β in the accumulated macrophages.

### Effect of LCCA injection into the trigeminal ganglion on the change in the head withdrawal threshold, Iba1, toll-like receptor 4, and IL-1RI expression

Previous studies indicated that accumulated macrophages and overexpression of toll-like receptor 4 and IL-1RI have important roles for the development of persistent and ectopic orofacial pain [21, 22, 27, 29, 33]; however, each interaction is still unknown. To examine the interaction among accumulated macrophages, toll-like receptor 4, and IL-1RI, the selective macrophage depletion agent LCCA was injected into the trigeminal ganglion. Then, the change in the head withdrawal threshold, the change in the expression of Iba1, toll-like receptor 4, and IL-1RI in trigeminal ganglion were examined on the rats with the operation of sham or tooth pulp exposure. The difference in effect of LCCA depending on the timing and frequency of injection was also investigated. The time course of the experimental procedure for the group receiving LCCA injection into the trigeminal ganglion immediately preceding head withdrawal threshold measurement in rats with tooth pulp exposure is shown in Fig. 3a. The time course of the experimental procedure for the groups receiving vehicle or LCCA injections into the trigeminal ganglion of rats with sham or tooth pulp exposure operation is shown in Fig. 3b. A significant decrease in head withdrawal threshold in response to heat (*p* = 0.001) or mechanical (*p* = 0.001) stimulation of the tongue was not reversed on day 1 after tooth pulp exposure in the group that received LCCA immediately preceding head withdrawal threshold measurement. However, a decrease in head withdrawal threshold in response to heat or mechanical stimulation of the tongue was significantly reversed on day 1 after tooth pulp exposure in rats that were administered daily injections of LCCA into the trigeminal ganglion compared to the rats that received vehicle (*p* = 0.001, Fig. 3c). Although a remarkable reduction of Iba1 expression was not observed in the group that received LCCA in the trigeminal ganglion immediately preceding head withdrawal threshold measurement in rats with tooth pulp exposure, daily administration of LCCA into the trigeminal ganglion markedly reduced macrophage accumulation on day 1 after tooth pulp exposure (Fig. 3d). No significant differences were observed in the mean percentages of fluorogold-labeled toll-like receptor 4-IR and IL-1RI-IR cells between vehicle- and LCCA-injected rats with tooth pulp exposure. Furthermore, compared with the daily vehicle- or LCCA-injected rats with tooth pulp exposure, no significant differences were observed in the mean percentages of fluorogold-labeled toll-like receptor 4-IR and IL-1RI-IR cells compared with the tooth pulp exposure rats group administered LCCA immediately preceding head withdrawal threshold measurement (Fig. 3e, f). The mean percentages of fluorogold-labeled toll-like receptor 4-IR, fluorogold-labeled IL-1RI-IR, and fluorogold-labeled toll-like receptor 4-IR+IL-1RI-IR cells in the rats with tooth pulp exposure administered of vehicle in the trigeminal ganglion were 43.1%, 10.9%, and 46.0% respectively (Fig. 3g). These results indicate two points. One is that although accumulation of macrophages in the trigeminal ganglion after tooth pulp exposure occurs, tongue heat or mechanical hyperalgesia is not involved in the upregulation of toll-like receptor 4 and IL-1RI coexpressing cells. The other is that daily LCCA and LPS-RS application before tooth pulp exposure is necessary to suppress accumulation of macrophage.

### Effect of LPS-RS injection into the trigeminal ganglion on the change in the head withdrawal threshold and IL-1RI expression

Evidence suggested that LPS-toll-like receptor 4 signaling potentiates IL-1β release and subsequently upregulates IL-1RI expression on the surface of macrophages in acute lung injury model [35]. Based on that previous result, we focused on the signal transduction between toll-like receptor 4 and IL-1R1 to investigate which is the preceding signal. To examine the signaling in the trigeminal ganglion following tooth pulp exposure, a selective toll-like receptor 4 antagonist LPS-RS was injected into the trigeminal ganglion. Then, the change in the head withdrawal threshold and the change in the expression of IL-1RI in trigeminal ganglion were examined on the rats with the operation of sham or tooth pulp exposure. The differences in the effect of LPS-RS depending on the timing and frequency of injection were also investigated. The time course of the experimental procedure for the group administered LPS-RS into the trigeminal ganglion immediately preceding head withdrawal threshold measurement of rats with tooth pulp exposure is shown in Fig. 4a. Furthermore, the time course of the experimental procedure for the groups administered vehicle or LPS-RS into the trigeminal ganglion of rats with sham or tooth pulp exposure procedure is shown in Fig. 4b. A significant decrease in head withdrawal threshold in response to heat (*p* = 0.001) or mechanical (*p* = 0.001) stimulation of the tongue was not reversed on day 1 after tooth pulp exposure in the group administered LPS-RS immediately preceding head withdrawal threshold measurement compared to tooth pulp exposure rats that received vehicle. However, a decrease in head withdrawal threshold in response to heat or mechanical stimulation of the tongue was significantly reversed on day 1 after tooth pulp exposure in rats that received daily injections of LPS-RS into the trigeminal ganglion compared to tooth pulp exposure rats that received vehicle and compared to the group administered LPS-RS immediately preceding head withdrawal threshold measurement (*p* = 0.001 each, Fig. 4c). The mean percentage of fluorogold-labeled IL-1RI-IR cells in LPS-RS-injected tooth pulp exposure rats was significantly decreased compared to vehicle-injected tooth pulp exposure rats (*p* = 0.001). The mean percentage of fluorogold-labeled IL-1RI-IR cells in LPS-RS-injected tooth pulp exposure rats was also significantly decreased compared with the group administered LPS-RS immediately preceding head withdrawal threshold measurement (*p* = 0.001). Furthermore, the mean percentage of fluorogold-labeled IL-1RI-IR cells in the group administered LPS-RS immediately preceding head withdrawal threshold measurement was significantly decreased compared with tooth pulp exposure rats that received vehicle (*p* = 0.003, Fig. 4d, e). These results indicate that toll-like receptor 4 signal is preceded on IL-1R1 signal and that daily LPS-RS injection before tooth pulp exposure is required for sufficient pharmacological effect.

### Effect of Hsp70 injection into the trigeminal ganglion on head withdrawal threshold and IL-1RI expression

In addition, we examined toll-like receptor 4–IL-1R1 signaling to determine which signal was upstream. To examine it, Hsp70, a selective agonist of toll-like receptor 4, was injected into the trigeminal ganglion of the naive rats. Then, head withdrawal threshold measurements and immunohistochemistry for IL-1RI were performed. The time course of the experimental procedure for groups administered vehicle or Hsp70 into the trigeminal ganglion is shown in Fig. 5a. The heat and mechanical head withdrawal thresholds were significantly decreased on day 1 following Hsp70 injection in the trigeminal ganglion relative to the pre-injection (day 7 vs. day 8, *p* = 0.001, Fig. 5b). The heat and mechanical head withdrawal thresholds were significantly decreased on day 1 following Hsp70 injection in the trigeminal ganglion relative to the rats injected with vehicle (vehicle vs. Hsp70, *p* = 0.001, Fig. 5b). The expression of fluorogold-labeled IL-1RI-IR cells in the trigeminal ganglion was increased in Hsp70-injected rats (Fig. 4c). The mean percentage of fluorogold-labeled IL-1RI-IR cells was significantly higher in Hsp70-injected rats relative to that in vehicle-injected rats (*p* = 0.001, Fig. 5d). Considering the data mentioned above (Fig. 4), these findings suggest that toll-like receptor 4 signaling is upstream in the toll-like receptor 4–IL-1R1 signaling.

### Effect of IL-1β injection into the trigeminal ganglion on head withdrawal threshold and TRPV1 expression

Previous report indicated that the satellite glial cells release IL-1β, and the IL-1β binds to IL-1RI on the trigeminal ganglion neuron, which subsequently facilitates TRPV1 expression on the same trigeminal ganglion neuron [29]. To examine the involvement of IL-1β–IL-1RI–TRPV1 signaling in the present study, head withdrawal threshold measurements and immunohistochemistry were performed following administration of IL-1β into the trigeminal ganglion of the naive rats. The time course of the experimental procedure for groups administered vehicle or IL-1β into the trigeminal ganglion is shown in Fig. 5e. The heat and mechanical head withdrawal thresholds were significantly decreased on day 1 following IL-1β injection in the trigeminal ganglion relative to the pre-injection (day 7 vs. day 8, *p* = 0.001, Fig. 5f). The heat and mechanical head withdrawal thresholds were significantly decreased on day 1 following IL-1β injection in the trigeminal ganglion relative to the rats injected with vehicle (vehicle vs. IL-1β, *p* = 0.001, Fig. 5f). The expression of fluorogold-labeled TRPV1-IR cells in the trigeminal ganglion was increased in IL-1β-injected rats (Fig. 4g). The mean percentage of fluorogold-labeled TRPV1-IR cells was significantly higher in IL-1β-injected rats relative to that in vehicle-injected rats (*p* = 0.001, Fig. 5h). These results indicate that IL-1β–IL-1RI signaling facilitates overexpression of TRPV1 on the trigeminal ganglion neuron, resulting in the development of heat and mechanical tongue hypersensitivity in the present study.

## Discussion

In our experiments, we reliably observed tongue heat and mechanical hypersensitivity following tooth pulp exposure. IL-1β production was observed in accumulated macrophages, with higher expression levels of toll-like receptor 4 and IL-1RI in trigeminal ganglion neurons innervating the tongue in rats with tooth pulp exposure. LCCA injection into the trigeminal ganglion significantly suppressed tongue hypersensitivity. However, no changes were observed in toll-like receptor 4 and IL-1RI expression levels in trigeminal ganglion neurons innervating the tongue. LPS-RS injection into the trigeminal ganglion after tooth pulp exposure significantly suppressed tongue hypersensitivity and decreased IL-1R expression in trigeminal ganglion neurons, whereas intra-trigeminal ganglion injection of recombinant Hsp70 enhanced tongue hyperalgesia and promoted IL-1RI expression in trigeminal ganglion neurons innervating the tongue in naive rats. Furthermore, intra-trigeminal ganglion injection of recombinant IL-1β also enhanced tongue hyperalgesia and promoted TRPV1 expression in trigeminal ganglion neurons innervating the tongue in naive rats. These results suggested that the neuron-macrophage interaction mediated through toll-like receptor 4 and IL-1RI signaling in the trigeminal ganglion neurons is important for the pathogenesis of ectopic and abnormal tongue pain sensation via TRPV1 following tooth pulp inflammation.

### Macrophage activation and its interaction with toll-like receptor 4 and IL-1RI in the tongue ectopic pain following tooth pulp exposure

Tooth pulp inflammation frequently causes persistent pain in the orofacial regions, which is termed ectopic orofacial pain, along with tooth pain [1–4]. Tooth pulp inflammation is accompanied by the enhancement of toll-like receptor 4 and IL-1β expression levels in trigeminal ganglion neurons [22]. In turn, IL-1β upregulates the expression of its own receptor IL-1RI [35]. In acute lung injury, LPS-toll-like receptor 4 signaling potentiates IL-1β release and subsequently upregulates IL-1RI expression on the surface of macrophages through MyD88 and NF-κB-dependent signaling [25]. Taken together, these evidences suggest that toll-like receptor 4 activation and subsequent upregulation of IL-1RI likely play an important role in ectopic orofacial pain associated with IL-1β expression in macrophages following tooth pulp inflammation.

In addition, orofacial inflammation or trigeminal nerve injury causes macrophage accumulation in the trigeminal ganglion [10, 12, 33]. Accumulated macrophages release various substances, including IL-1β, that enhance neuronal excitability [13–15, 36]. Conversely, macrophage depletion by LCCA significantly suppresses macrophage accumulation, which reduces TNFα release, finally resulting in the recovery of the mechanical hypersensitivity of whisker pad skin following inferior alveolar nerve transection [33]. In the present study, we observed a significant increase in the heat and mechanical tongue hypersensitivity following tooth pulp exposure. The tooth pulp exposure enhanced the expression of toll-like receptor 4 and IL-1RI in trigeminal ganglion neurons and accelerated the release of IL-1β subsequent to macrophage accumulation in the trigeminal ganglion. Remarkable changes in head withdrawal threshold and macrophage accumulation were not observed on day 1 after tooth pulp exposure in the group administered LCCA into the trigeminal ganglion immediately preceding head withdrawal threshold measurement but were observed in the group that received daily pre-injections before tooth pulp exposure. Notably, although the depletion of activated macrophages attenuated the tooth pulp exposure-induced tongue hyperalgesia, toll-like receptor 4 and IL-1RI expression levels were not altered, indicating macrophages accumulation are not involved in the upregulation of toll-like receptor 4 and IL-1RI expression. Furthermore, remarkable changes in head withdrawal threshold was not observed on day 1 after tooth pulp exposure in the group administered LPS-RS into the trigeminal ganglion immediately preceding head withdrawal threshold measurement but was observed in the group that received daily pre-injections before tooth pulp exposure. The IL-1RI expression was significantly decreased in the group that received daily pre-injections of LPS-RS before tooth pulp exposure compared to the group that received vehicle. On the other hand, interestingly, significant decrease in the IL-1RI expression was also observed in the group administered LPS-RS immediately preceding head withdrawal threshold measurement, compared to the group that received daily pre-injections of vehicle before tooth pulp exposure. This is may be because it takes some time from the injection of the drug to the perfusion, and the effect of the drug appeared to some extent during that period.

Together, these results indicate that daily LCCA and LPS-RS application before tooth pulp exposure is necessary to suppress macrophage accumulation and toll-like receptor 4 signaling. These results also suggest that toll-like receptor 4 signal is preceded on IL-1R1 signal and that toll-like receptor 4-IL-1RI signaling mediated by IL-1β released from accumulated macrophages likely plays an important role in the pathogenesis of tongue hyperalgesia following tooth pulp exposure.

### Hsp70 involvement in the enhancement of IL-1RI expression in trigeminal ganglion neurons

Hsp70 is expressed in the tooth pulp following pulpal trauma, heat stress, or inflammation [21, 37, 38]. Recombinant-Hsp70 applied to the tooth pulp is axonally transported to the trigeminal ganglion and released from trigeminal ganglion neurons into the extracellular space [21]. The secreted Hsp70 binds to toll-like receptor 4 of the trigeminal ganglion neurons innervating the tongue, leading to tongue hypersensitivity [21]. In the present study, intra-trigeminal ganglion injection of recombinant Hsp70 induced tongue-mechanical and heat hypersensitivity accompanied by increases IL-1RI expression. Together, these data suggested that Hsp70 expression in the tooth pulp following tooth pulp exposure likely contributes to IL-1RI upregulation through toll-like receptor 4 activation in the trigeminal ganglion, resulting in the pathogenesis of tongue hypersensitivity. Moreover, the present results demonstrated that the elimination of IL-1β through LCCA-mediated macrophage depletion did not affect toll-like receptor 4 and IL-1RI expression. On the other hand, although LPS-RS downregulated IL-1RI expression, Hsp70, conversely, upregulated it. These results also suggested that the regulation of IL-1RI expression occurs downstream of toll-like receptor 4 signaling.

### IL-1β involvement in the upregulation of TRPV1 expression in trigeminal ganglion neurons

TRPV1 expression is potentiated in trigeminal ganglion neurons following peripheral inflammation or nerve injury, resulting in the development of mechanical and/or thermal hypersensitivity [39, 40]. The activation of IL-1RI by IL-1β facilitates TRPV1 expression and promotes neuronal hyperexcitability [27, 29, 41]. In the present study, intra-trigeminal ganglion injection of IL-1β promoted tongue hypersensitivity to mechanical and heat stimuli and augmented TRPV1 expression. Thus, these results suggested that TRPV1 upregulation is involved in the occurrence of tongue hypersensitivity subsequent to the activation of IL-1RI by the IL-1β expressed in the trigeminal ganglion.

## Conclusions

**Fig. 6:**
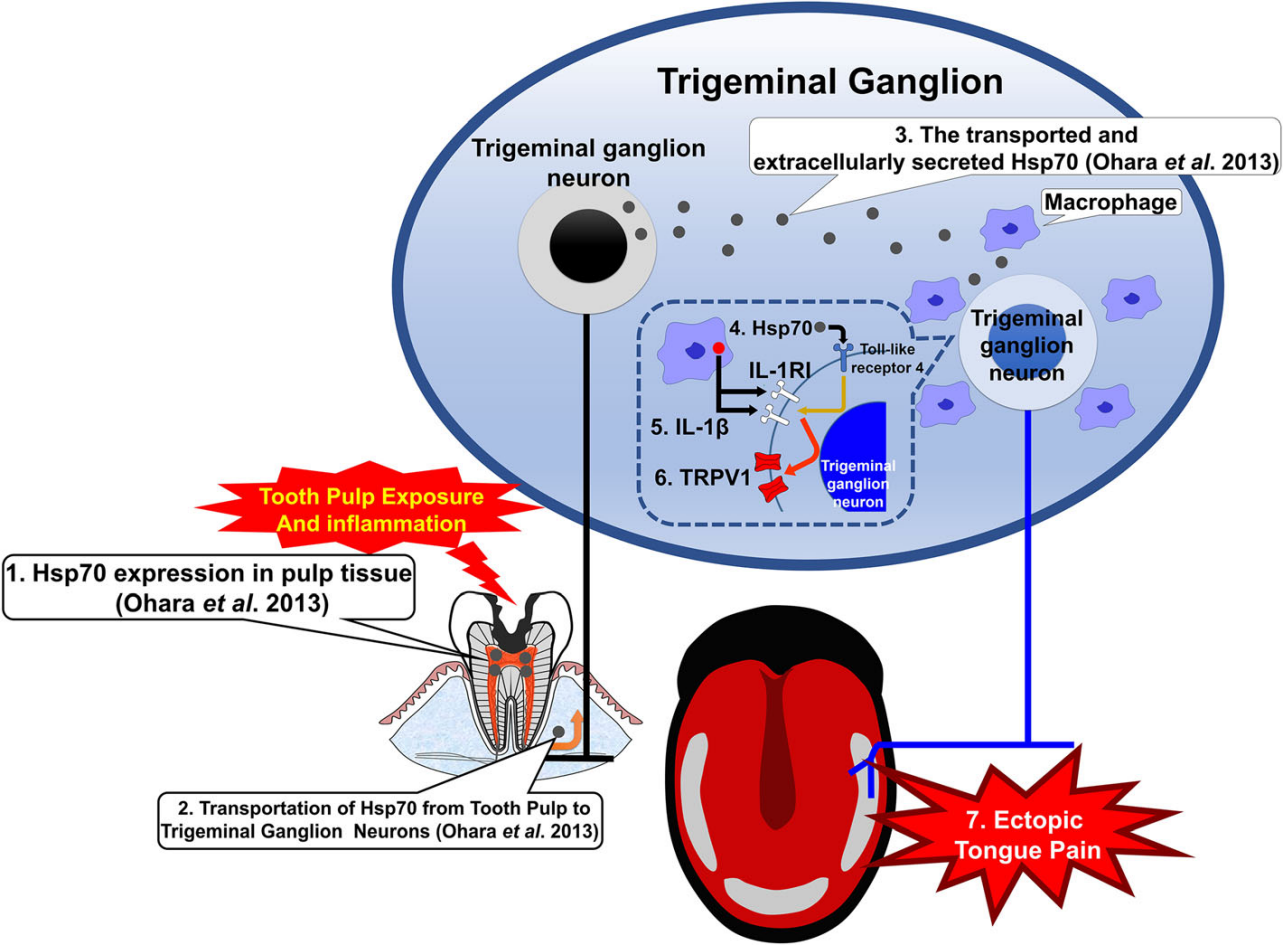
Schematic results of the current study. (1) Hsp70 is expressed in the pulpal tissue following tooth pulp exposure. (2) Hsp70 is axonally transported from the inflamed tooth pulp of the lower first molar to the trigeminal ganglion. (3) Transported Hsp70 is released into the intercellular space in the trigeminal ganglion. (4) Hsp70 binds to the toll-like receptor 4 of trigeminal ganglion neurons innervating the tongue and promotes IL-1RI expression. (5) Simultaneously, the accumulated macrophages following tooth pulp inflammation generate and release IL-1β in the extracellular space in the trigeminal ganglion. Larger amounts of IL-1β bind to the larger number of IL-1RI of trigeminal ganglion neurons innervating the tongue. (6) The IL-1β/IL-1RI signaling facilitates TRPV1 expression. (7) Enhanced expression of TRPV1 on the tongue-innervating trigeminal ganglion neurons promotes neuronal excitability and results in mechanical and heat hypersensitivity of the tongue

Our experiments suggest the following mechanism underlying tongue hypersensitivity after tooth pulp exposure (Fig. 6). Mandibular first tooth pulp exposure induces Hsp70 expression in the pulpal tissue, which is axonally transported from the lower first molar to the trigeminal ganglion and released into the intercellular space in the trigeminal ganglion. Subsequent Hsp70 binding to toll-like receptor 4 of the trigeminal ganglion neurons innervating the tongue promotes IL-1RI expression. Simultaneously, accumulated macrophages generate and release IL-1β in the trigeminal ganglion intercellular space following tooth pulp exposure, which binds to the overexpressed IL-1RI of trigeminal ganglion neurons innervating the tongue. The IL-1β-IL-1RI signaling, in turn, facilitates TRPV1 expression in the tongue-innervating trigeminal ganglion neurons. Finally, augmented levels of TRPV1 enhance trigeminal ganglion neuronal excitability, resulting in mechanical and heat hypersensitivity of the tongue. Our results indicate that the development of ectopic tongue hypersensitivity induced by tooth pulp inflammation is associated with the accumulation of macrophages in the third branch region in the trigeminal ganglion, IL-1β production by macrophages, and increased expression levels of toll-like receptor 4 and IL-1RI. The results of this study revealed that macrophage activation and toll-like receptor 4/IL-1RI/TRPV1 signaling play important roles in the development of abnormal pain, and continued research will further elucidate the pathogenesis of abnormal pain in the maxillofacial region. Our present research may contribute to the establishment of new therapeutic and diagnostic methods and the development of therapeutic agents.

## Data Availability

All data generated or analyzed during this study are included in this published article.

## Abbreviations

ANOVA: Analysis of variance
Hsp70: Heat shock protein 70
IL-1β: Interleukin-1β
IL-1RΙ: Interleukin-1 type I receptor
IR: Immunoreactive
LCCA: Liposomal clodronate Clophosome-A
LPS-RS: Lipopolysaccharide from *Rhodobacter sphaeroides*
SEM: Standard error of the mean
TRPV1: Transient receptor potential vanilloid 1
